# A High-Performance Ultraviolet Optical Sensing System for Rotating Detonation Extreme Combustion

**DOI:** 10.3390/s26103248

**Published:** 2026-05-20

**Authors:** Wen Dai, Yingchen Shi, Junhui Ma, Mingyang Bu, Lingxue Wang, Qiaofeng Xie, Haocheng Wen, Bing Wang

**Affiliations:** 1 School of Aerospace Engineering, Tsinghua University, Beijing 100084, China; daiw21@mails.tsinghua.edu.cn (W.D.); shi-yc21@mails.tsinghua.edu.cn (Y.S.);; 2 MIIT Key Laboratory of Complex-Field Intelligent Exploration, School of Optics and Photonics, Beijing Institute of Technology, Beijing 100081, China; 3 Institute for Aero Engine, Tsinghua University, Beijing 100084, China

**Keywords:** ultraviolet optical sensing, rotating detonation extreme combustion, multi-spectral observation, high spatiotemporal, chemiluminescence

## Abstract

Extreme combustion features strong unsteadiness, heterogeneity and multi-physics coupling, which is of great significance for advanced propulsion systems. High-performance sensing of such extreme combustion flow fields is critical to revealing physical mechanisms and capturing fine flow structures. However, it faces severe challenges: rich multi-band spectral characteristics require multi-spectral observation; ultra-transient processes demand high-frequency imaging; and high-performance photoelectric enhancement is necessary under short gate width and high frame rates. To solve these problems, this study developed a high-performance ultraviolet optical sensing system (HUOSS), which achieves megahertz-level imaging at a 1608 × 1104 full-frame resolution and provides a 10^7^ electron gain in the ultraviolet band. The HUOSS has been applied to chemiluminescence sensing of a hydrogen/ammonia-air rotating detonation as a representative extreme combustion system. Based on the analysis of representative influencing factors (e.g., the transmission characteristic of the bandpass filter and the intensifier gate width) in the HUOSS, the filter transmission loss and its influence on the gate width settings have been revealed. From the chemiluminescence sensing images captured in the experiments, the fine structure and evolution of detonation waves have been clearly identified, verifying the high-speed imaging capability. Furthermore, simultaneous OH* and NH* multi-spectral observation has been realized, and the effects of ammonia addition have been analyzed, validating the multi-spectral diagnostic capacity of the system. This study provides an effective diagnostic method for extreme transient combustion research, and comprehensively verified the multi-spectral, extremely transient and high signal-to-noise ratio sensing capabilities of this system.

## 1. Introduction

With the ever-increasing demands for higher power density, energy conversion efficiency, and operating limits of power devices in aerospace, transportation, energy generation and other fields, the combustion processes in energy and power systems are gradually evolving toward extreme conditions. The core characteristics of these combustion processes include strong unsteadiness, significant spatial non-uniformity, and the intense coupling of multi-physics. Traditional combustion theories and control strategies can hardly accommodate the complex behaviors of such extreme combustion scenarios, which has become a critical bottleneck restricting performance breakthroughs in advanced power devices. For example, as the core power source for ground transportation, high-pressure and high-speed internal combustion engines exhibit extreme combustion characteristics. To achieve high power density and fuel economy, engine speed and mean effective pressure are elevated to the limits of structural integrity [1]. The combustion process couples atomization, mixing, and chemical reaction on millisecond timescales, and significant concentration gradients form under the influence of jet flow, swirl flow, and combustor geometry [2,3]. Furthermore, in scramjet engines, extreme combustion is governed by strong dynamic variations in inflow conditions and their deep coupling with chemical reactions. Since ramjet engines involve numerous complex physical processes such as turbulent jet flow, mixing layers, acoustic pressure fluctuations, recirculation zones, combustion flow inside nozzles, and entropy waves, multiple inherent mechanisms can induce combustion instabilities in the ramjets [4]. This further exacerbates the extremity and complexity of the ramjet combustion characteristics.

In addition, detonation represents another form of extreme combustion, which is characterized by the coupled supersonic propagation of shock waves and reaction fronts [5]. Compared to pulsed detonation and standing detonation, rotating detonation has the advantage of being able to maintain continuous detonation with a single ignition and strong adaptability to inlet flows, which has attracted extensive attention in energy utilization and propulsion [6,7,8,9,10,11,12]. But multiple instability phenomena exist in the rotating detonation combustor, including chaotic instability, waxing and waning instability, transient mode switching instability, and longitudinal pulsed detonation instability [13], which directly affect the propagation of detonation waves and overall combustion performance. In addition, the flow field inside the rotating detonation combustor is inherently unsteady. Detonation waves generally propagate at a velocity of 1000–2000 m/s [14], posing great challenges to experimental measurements.

The combustion processes of the energy and power devices mentioned above all exhibit distinct extreme characteristics, which pose numerous challenges to the investigation of combustion mechanisms and engineering applications. The strongly transient, high-temperature, high-pressure, and complex multi-physics coupling characteristics of extreme combustion flows render traditional contact measurement methods (e.g., thermocouples, pressure probes, etc.) subject to problems including large measurement disturbances, insufficient spatiotemporal resolution, and vulnerability to damage in high-temperature and high-pressure environments. These methods are unable to accurately capture the dynamic evolution of extreme combustion flows. Therefore, optical sensing technologies have become the core approach for the research and diagnosis of extreme combustion flows [15,16,17,18]. They possess unique advantages such as non-intrusiveness, high spatiotemporal resolution, and the capability of simultaneous multi-parameter measurement. They can not only avoid disturbances to the combustion flow field but also precisely capture the transient characteristics and spatial distribution of combustion flows under extreme conditions.

Among the commonly used optical measurement techniques for extreme combustion flows, the ultraviolet (UV) spectral band occupies a central role in extreme combustion diagnostics [19,20] owing to its distinct advantages: relatively strong penetrability, characteristic interactions with key reactive radicals (e.g., OH*, NH*, NO*, etc.) during combustion, and low susceptibility to interference from flame self-luminescence. Its typical applications mainly include the following two categories. The first is UV planar laser-induced fluorescence (UV-PLIF). It employs a tunable laser as the light source. When the laser illuminates the combustion flow field, the target species are excited to emit fluorescence, which is then imaged by a detector. This technique enables high-precision measurements of the two-dimensional spatial distribution of reactive species, flame front structure, and heat release rate in the combustion flow field. It has been successfully applied to investigations such as in-cylinder concentration measurements in internal combustion engines [21], combustion characteristics in scramjets [18], and the flow dynamics of detonation waves [17,22,23,24]. The second is the UV chemiluminescence imaging technique. As a passive optical measurement method, its core principle is that key reactive radicals undergo electronic transitions during extreme combustion and spontaneously emit characteristic spectra in the UV band. By capturing such chemiluminescence signals with a high-sensitivity UV camera, dynamic visualization and qualitative or quantitative analysis of the flame front, reaction zone distribution and combustion intensity in the combustion flow field can be achieved. Compared with UV-PLIF, the key advantages of this method lie in its simple structure, non-intrusiveness and cheaper [25], which make it suitable for long-term online monitoring in extreme combustion scenarios. Therefore, it has been widely used in extreme combustion measurements, as shown in Table 1.

**Figure 1:**
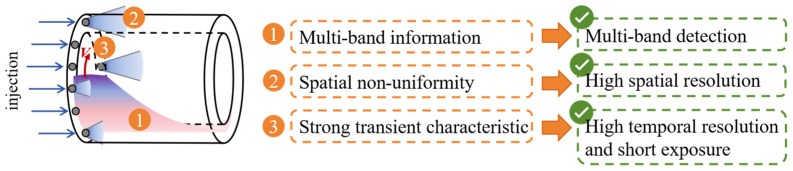
Difficulties in optical measurements of extreme combustion.

Although optical measurement technology has become the core approach for the study of extreme combustion flows, it still encounters three major difficulties, as shown in Figure 1. First, extreme combustion is accompanied by abundant spectral information across multiple bands. The realization of multi-spectral simultaneous observation is challenging, yet it serves as a prerequisite for obtaining comprehensive multi-physical field data. Second, the strongly transient characteristic and spatial non-uniformity of extreme combustion impose stringent requirements on the high spatiotemporal resolution of the optical measurement. Third, the dual constraints of an ultra-short gate width (GW) and a megahertz-level imaging frame rate dictate that the optical measurement must rely on high-performance optoelectronic intensification equipment to ensure effective signal-to-noise ratio (SNR) and high imaging sensitivity.

Therefore, in response to the above limitations, we developed a high-performance UV optical sensing system (HUOSS) with high spatiotemporal resolution and high gain. The core of this system relies on an intensified complementary metal oxide semiconductor (ICOMS) based on a high-quantum-efficiency UV intensifier, together with an established sensing cluster and a high-precision triggering system, enabling it to achieve the multi-band detection, high spatiotemporal resolution, and high SNR required for extreme combustion flow scenarios. We applied this HUOSS to the chemiluminescence sensing of rotating detonation flow fields of hydrogen/ammonia-oxygen fuels, aiming to fully validate the capability of the HUOSS for extreme combustion flow field sensing. A theoretical analysis has been conducted to characterize the influence of the bandpass filter and gate width settings on the sensing performance of the system for detonation chemiluminescence. Based on the theoretical analysis results, a series of experiments has been carried out, and high-quality UV optical sensing images have been acquired. Clear detonation wave structures, detonation wave evolution processes, and multi-band imaging results have been captured, which fully validate the measurement and the sensing performance of the system. This system is also expected to be applied to the measurement of other extreme combustion processes.

## 2. High-Performance Ultraviolet Optical Sensing System

According to the testing requirements for extreme combustion flow fields, we developed the UV optical sensing system with high spatiotemporal resolution and high gain based on the previous work [30,31]. But different from our previous work, which mainly focused on the preliminary imaging verification of a single ICMOS prototype for rotating detonation diagnostics, the present study further extends the developed system into a modular HUOSS for extreme combustion research. In addition to the collaborative architecture of multiple ICMOS units, a Photomultiplier tube (PMT)-triggered synchronized imaging strategy was introduced to improve the timing controllability and repeatability of ultra-transient sensing. Therefore, the focus of this work is not only on a high-speed imaging demonstration, but also on the establishment of a systematic sensing framework integrating synchronized triggering, multi-spectral collaboration, and high-spatiotemporal-resolution diagnostics. This chapter presents the core design philosophy and the detailed composition of the system.

### 2.1. Design of the ICOMS Unit

**Figure 2:**
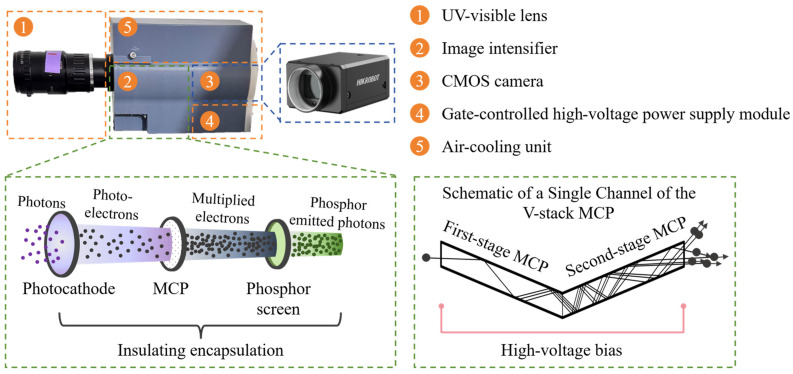
Schematic diagram of the self-developed ultraviolet high-gain ICMOS.

The core of the HUOSS relies on the high performance ICMOS unit. The schematic diagram of the self-developed ultraviolet high-gain ICMOS is presented as shown in Figure 2. The ICMOS unit mainly consists of a UV-visible lens (AZURE-7538UV, AZURE PHOTONICS Co., Ltd., Fuzhou, China), an image intensifier, a complementary metal-oxide-semiconductor (CMOS) camera (MV-CA017-10GM, Hikvision Digital Technology Co., Ltd., Hangzhou, China), a gate-controlled high-voltage power supply module, and an air-cooling unit. The overall size of ICOMS unit is approximately 60 mm × 100 mm × 125 mm. During operation, the weak optical signal is first collected by the UV-visible lens and then transmitted to the image intensifier, where photon-to-electron conversion occurs at the photocathode. The generated photoelectrons are subsequently multiplied by the microchannel plate (MCP) and then converted back into visible photons at the phosphor screen. Finally, the photons emitted from the phosphor screen are recorded by the CMOS sensor to complete the image acquisition.

**Figure 3:**
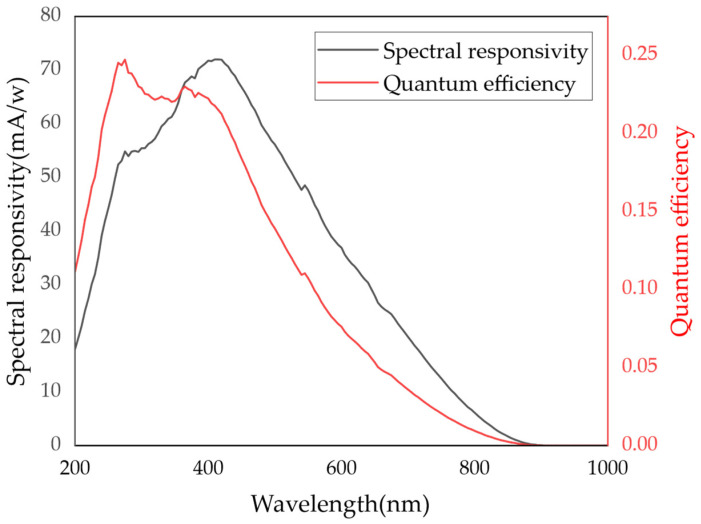
The measured spectral responsivity and quantum efficiency of the photocathode employed in the ICMOS.

The image intensifier is a key component of the ICMOS unit for weak-light detection and electronic amplification, and it mainly consists of a UV photocathode, an MCP, and a phosphor screen. By applying either a positive or negative voltage between the photocathode and the MCP through a high-voltage module driven by a high-frequency digital delay pulse generator, the image intensifier can be switched on or off, thereby enabling gated imaging. Within the image intensifier, the weak chemiluminescence radiation is first converted into an electronic signal through photoemission at the photocathode. After electron multiplication in the MCP, the signal is reconverted into a detectable optical signal by the phosphor screen and subsequently captured by the CMOS array. The internal operating mechanism of the image intensifier is further illustrated in the enlarged view in the lower left corner of Figure 2. The spectral responsivity and measured quantum efficiency of the photocathode used in the ICMOS are presented in Figure 3, indicating a favorable photoelectric response capability in the UV-visible spectral range.

In the ICMOS unit, the gain of the image intensifier is further improved through the optimization of both the MCP structure and the coupling method. In terms of the MCP configuration, a two-stage V-stack structure composed of two MCPs connected in series is adopted to further increase the overall electron gain of the image intensifier [32]. In this configuration, the two MCPs are arranged with a certain bias angle, such that the electrons multiplied by the first-stage MCP enter the second-stage MCP and undergo further secondary electron emission, thereby forming a cascaded electron multiplication process. The single-channel structure of the double-layer V-stack MCP is illustrated in the lower right corner of Figure 2. When electrons enter the first-stage MCP, they experience continuous secondary electron emission along the channel wall and are further amplified in the second-stage MCP, resulting in a substantially enhanced electron multiplication. Compared with a single MCP, the double V-stack structure significantly increases the number of multiplication stages and thus provides a higher output gain under a nanosecond integration time. Meanwhile, the opposite bias-angle arrangement of the two MCPs can suppress ion feedback and electron backscattering to a certain extent, thereby improving both the operational stability and signal-to-noise performance of the device.

After achieving a high electron gain through the double-MCP structure, the efficient coupling of the visible image emitted from the phosphor screen to the sensor surface remains essential. To reduce the optical energy loss associated with conventional relay-lens coupling and to improve system compactness, a direct coupling scheme is adopted, in which the output end of the image intensifier is closely coupled to the CMOS sensor. This configuration increases the effective signal intensity reaching the sensor surface under limited photon-flux conditions, thereby facilitating the acquisition of distinguishable images under a nanosecond integration time. The signal level delivered to the CMOS sensor through direct coupling can be approximately 5–10 times higher than that achieved using conventional lens coupling [33], which significantly alleviates the limitation imposed by CMOS readout noise in weak-signal detection. In addition, direct coupling offers the advantages of a compact structure, high mechanical stability, and considerable potential for further system miniaturization.

### 2.2. Concepts of Sensing Cluster Design

**Figure 4:**
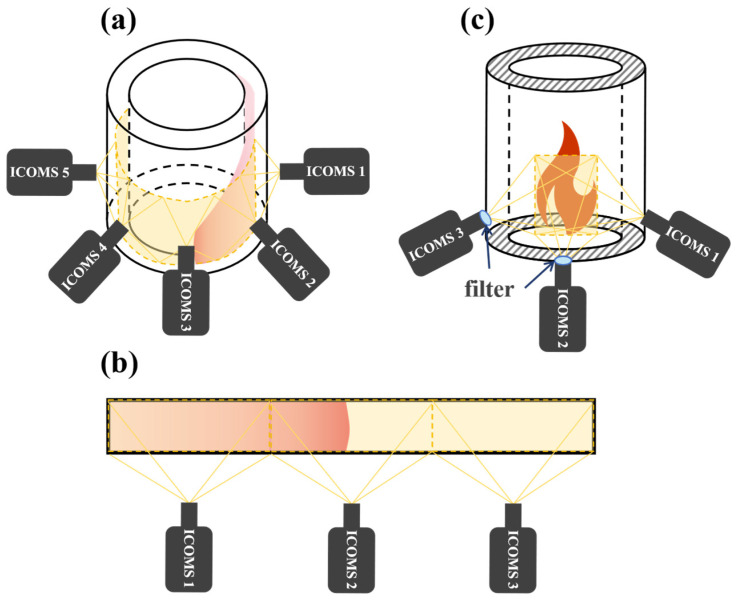
Three Design Concepts for Sensing Cluster of HUOSS: (**a**) 3D sensing; (**b**) Full-frame high-framerate and multi-band sensing; (**c**) Large-area planar sensing.

Although a single ICMOS unit features high sensitivity and high gain, it is limited by the CMOS sensor we currently used, which achieves a maximum frame rate of only 68.5 fps at a 1608 × 1104 full-frame resolution (i.e., the frame rate and resolution could be further improved by upgrading to higher-performance CMOS sensors; however, this will lead to a significant increase in the cost of the ICMOS unit). As a result, it is difficult to perform high-spatiotemporal-resolution measurements independently, and it cannot acquire multi-band spectral information simultaneously. Therefore, based on the compact size and relatively low cost of the ICMOS unit described in Section 2.1, we propose three design concepts for the sensing cluster of the HUOSS, as shown in Figure 4.

As shown in Figure 4a, when multiple ICMOS units cover the flow field across 360°, capturing images with these ICMOS units simultaneously allows for the acquisition of three-dimensional data of the combustion flow field, and these obtained images can be directly used for the three-dimensional reconstruction of the flame. Owing to the relatively low cost of the ICMOS unit, a large-angle field-of-view coverage can be employed, avoiding the degradation of imaging accuracy caused by using reflecting mirrors to reduce the number of ICMOS units [34]. For large-scale planar flame combustion flow fields (e.g., detonation tubes), a linear array as illustrated in Figure 4b can be adopted. By triggering the ICMOS units to image synchronously, full-field flow-field images can be obtained. Finally, when multiple ICMOS units are focused on the same imaging area, a high-frame-rate imaging performance similar to that of high-speed ICMOS units can be achieved by controlling the interval time between the ICMOS units and performing image correction, as shown in Figure 4c. Compared to high-speed ICMOS units, this scheme preserves full-frame resolution at high speeds, therefore enabling finer capture of the detailed flow structures. Furthermore, in the configuration of Figure 4c, bandpass filters of different spectral bands can be mounted in front of different ICMOS units. By capturing images simultaneously, multi-band sensing can be realized, which can be applied to the analysis of different combustion species.

### 2.3. The Specific Design of HUOSS in This Study

**Figure 5:**
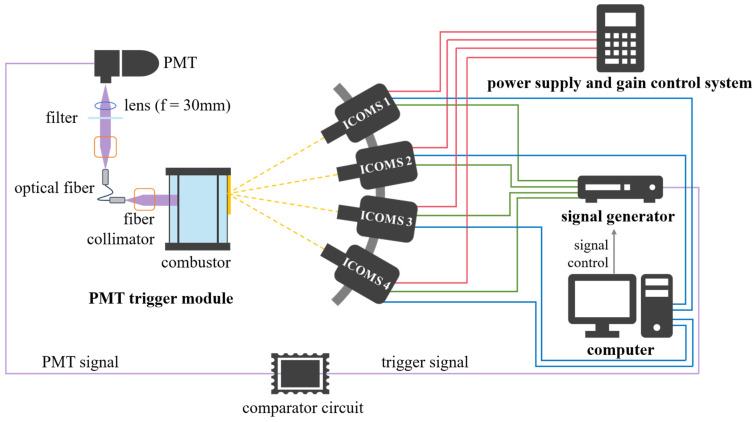
Schematic diagram of HUOSS in the current study.

In this study, we applied the developed HUOSS to radical chemiluminescence sensing in the rotating detonation combustion system, to validate its sensing performance in extreme combustion scenarios. As shown in Figure 5, four ICMOS units are integrated and the concept in Figure 4b is adopted in the present study. Each ICMOS unit operates independently and can work cooperatively, thereby forming the HUOSS for extreme combustion flow field sensing. In addition to the four ICMOS units, the HUOSS also includes a self-developed power supply and gain control system, a high-frequency signal generator (ASG24100, CIQTEK Technology Co., Ltd., Hefei, China), a PMT-trigger module and a computer. Among them, PMTs are typical high-sensitivity single-photon detectors for weak-light scenarios [35], which have been successfully applied to capture time-resolved OH chemiluminescence signals in rotating detonation combustion [36,37]. The PMT used in this study is model PMTH-S1-CR131A from the Zolix Instruments Co., Ltd. (Beijing, China), and its optical path diagram is shown on the left side of Figure 5. The front end of the optical fiber is aligned with the target area of the combustor, and a fiber collimator and a light shield are installed at the fiber tip to suppress stray light interference from other regions as much as possible, ensuring that the received signal mainly originates from the targeted area. In this way, a PMT time-domain signal characterizing the periodic motion of the wave front can be obtained. Subsequently, the output signal of the PMT is connected to the self-developed comparator circuit with a preset trigger threshold. When the output voltage of the PMT exceeds the set value, the comparator module outputs a trigger signal to activate the ICMOS units, realizing synchronized imaging corresponding to the arrival time of the wave front. The trigger thresholds used in this study were determined statistically from repeated measurements under each operating condition. The trigger threshold must be set such that the frequency of PMT signal peaks meeting or exceeding the threshold is greater than or equal to the acquisition frequency of the ICMOS unit in the HUOSS (i.e., 68.5 fps in this study).

**Figure 6:**
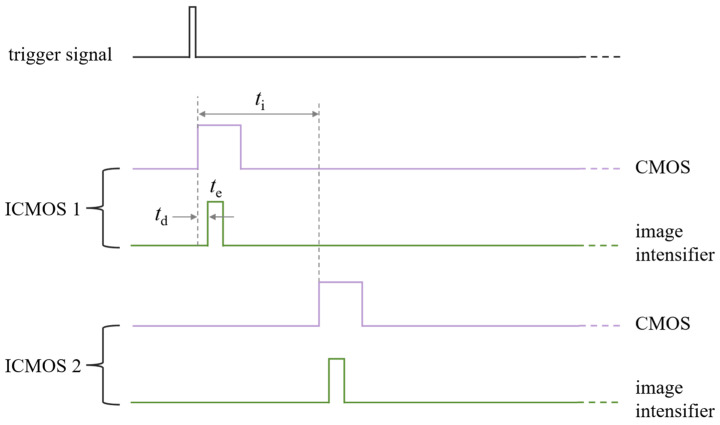
Timing sequence of HUOSS.

To achieve high-frame-rate measurements at full-frame resolution, the four ICMOS units are required to expose sequentially within one imaging cycle, and their imaging timings must be strictly controlled by the high-frequency signal generator. As shown in Figure 6, the timing sequence between ICMOS 1 and ICMOS 2 is presented. The timing for ICMOS 3 and ICMOS 4 follows the same pattern.

Here, *t*_e_ is the GW of the image intensifier, *t*_d_ is the delay time between the image intensifier and the CMOS camera, and *t*_i_ is the interval time between two adjacent ICMOS units. The delay time *t*_d_ is set to ensure that the intensified optical signal output by the image intensifier can be captured by the CMOS sensor within the same imaging cycle; in this work, it is set to 50 μs. Therefore, when *t*_e_ and *t*_i_ of the four ICMOS units are kept consistent, high-frame-rate measurements at a full-frame resolution can be realized, and four consecutive frames can be obtained in the same cycle, with an equivalent frame rate of 1/*t*_i_. In addition, to meet the requirements of multi-band sensing, optical filters of different bands can be installed in front of the lenses of different ICMOS units. By setting *t*_i_ of the four ICMOS units to 0, synchronous multi-band measurements can be accomplished.

## 3. Analysis of Representative Influencing Factors in Sensing System

For the chemiluminescence sensing of rotating detonation combustion flow fields, the parameter matching of the ICMOS-based optical sensing system directly governs the reliability of the imaging results in practical experiments. The bandpass filter and intensifier GW are the two most critical, strongly coupled parameters across the full system chain: the former guarantees the spectral specificity of the sensing signal, and the latter is the key parameter balancing the imaging SNR and time-integration effects. This chapter takes the widely used OH* chemiluminescence in hydrogen combustion as an example, systematically analyzes the mechanism by which these two parameters affect the imaging sensing performance, and provides theoretical support for the optimization of experimental parameters.

### 3.1. Influence of Bandpass Filter

The selection of the bandpass filter is critical to the optical sensing system, as it must balance out-of-band interference rejection with the preservation of the spectral intensity in the target waveband for practical experiments. Focusing on OH* chemiluminescence sensing, this section first characterizes the full-spectrum properties of the hydrogen-fueled rotating detonation flow fields, and then quantitatively assesses the matching performance between the selected bandpass filter and the OH* chemiluminescence emission over a wide temperature range.

**Figure 7:**
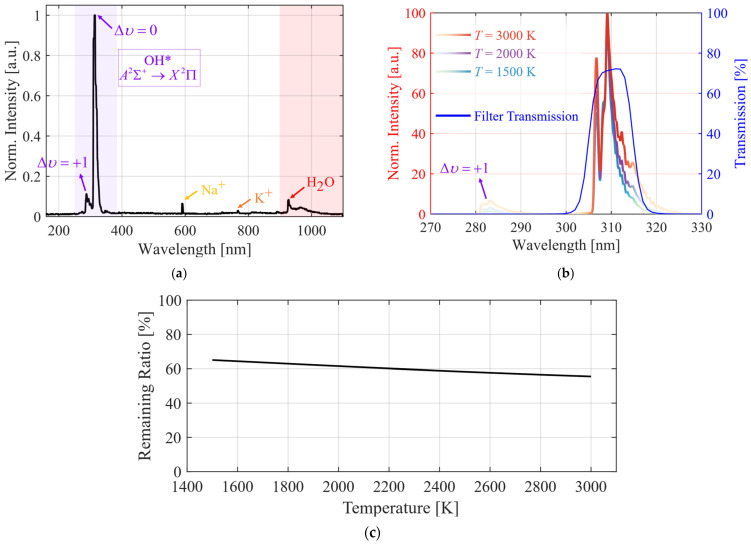
Spectral characteristics of rotating detonation flow field and matching performance between filter and OH* chemiluminescence spectrum: (**a**) full-band spectrum of hydrogen-fueled rotating detonation flow field [30]; (**b**) simulated OH* chemiluminescence spectrum at different temperature and transmission profile of used bandpass filter; (**c**) remaining ratio of total OH* chemiluminescence intensity through the filter at different temperature conditions.

Figure 7a shows the experimentally measured averaged spectrum of the hydrogen-fueled rotating detonation flow field [30]. The A^2^Σ^+^→X^2^Π electronic transition of excited OH* is clearly the dominant emission source in the current combustion system, with its Δυ = 0 vibrational band (i.e., the theoretical central wavelength ≈ 309 nm) providing the highest SNR and the most stable characteristic signature of the combustion process. Additional features visible in the spectrum include flame emission from alkali metal impurities (i.e.,: Na^+^ at 590 nm and K^+^ at 770 nm, likely from high-pressure gas cylinders or combustor hardware) and H_2_O vibrational-rotational near-infrared emission spanning 900–1000 nm. OH* exhibits strong signal intensity, high selectivity, and high coincidence with the heat release zone in rotating detonation combustion flow fields of hydrogen–air mixtures, making it an ideal optical tracer for studying extreme combustion flows. In this study, a UV bandpass filter (ZBPA310, Asahi Spectra Co., Ltd., Tokyo, Japan, central wavelength length (CWL): 310 ±2 nm, full width at half maximum (FWHM): 10 ± 2 nm) was used to isolate OH* chemiluminescence.

Figure 7b presents the OH* A→X chemiluminescence spectrum at varying temperatures calculated by LIFEBASE v1.5 [38], overlaid with the transmission curve of the filter used in this work. The color depth of the spectrum profile denotes the relative magnitude of the filter transmission at each wavelength. The central wavelength of the main OH* chemiluminescence peak is consistently anchored near 309 nm, with no observable peak shift. Nevertheless, rotational broadening increases marginally with temperature, accompanied by a rise in the intensity of the Δ*υ* = +1 vibrational band. Given the fixed transmission profile of the filter, this leads to a temperature-dependent variation in the total transmitted OH* chemiluminescence intensity. Figure 7c quantifies the remaining ratio of the total OH* chemiluminescence intensity transmitted through the filter as a function of temperature. For typical combustion conditions, the transmitted intensity ratio remains around 60%, and decreases with rising temperature under the combined influence of rotational broadening and Δ*υ* = +1 band intensity changes, with an overall variation of approximately 17%.

In conclusion, fixed-bandwidth bandpass filters present an inherent trade-off for chemiluminescence detection across wide operating conditions: narrowband filters risk truncating the target signal and degrading the SNR, whereas wideband filters have poor spectral selectivity and are prone to interference from other radical emissions (e.g., NH*/NO* chemiluminescence at around 336/226 nm, respectively) or broadband background noise. Furthermore, the temperature-dependent transmission of the total OH* chemiluminescence intensity through the filter also challenges measurement accuracy. These adverse effects can be mitigated in practice via careful filter selection. For the filter used in this work, near-peak transmittance is achieved at the OH* emission peak, with basically full coverage of the Δ*υ* = 0 vibrational band. Theoretical assessments confirm that the variation in the total transmitted OH* intensity is small across the wide temperature range of the experiments, making it well suited for the intended measurements.

### 3.2. Influence of Gate Width

Rotating detonation combustion flow fields are highly transient, rendering the multi-exposure averaging approach for SNR enhancement in conventional (quasi-) steady combustion unsuitable. The GW of the sensing system is thus a critical parameter here. An overly narrow GW causes insufficient target signal accumulation, a sharp SNR reduction, and severe noise interference, while an excessively wide GW drastically aggravates the time-integration effect and prevents the accurate capture of the transient detonation wave structure. Accordingly, selecting a proper GW to balance SNR preservation and the mitigation of time-integration effects is a core prerequisite for high-quality OH* chemiluminescence sensing of rotating detonation flow fields, and it is the primary focus of this section. Through theoretical calculations, this section assesses the cumulative OH* chemiluminescence signal under varying sensing-system GWs, to provide a reference for GW selection in practical experiments. In this study, the “sensing-system GW” specifically refers to the intensifier GW.

For the numerical setup, the Zel’dovich–von Neumann–Döring (ZND) structure of the stoichiometric H_2_-air detonation was solved by using SDToolbox v2021, considering the rotating detonation sensing scenario in this work. As a classical theory for one-dimensional steady planar detonation waves, the ZND model was the first to incorporate finite-rate chemical reactions compared to prior theories, enabling a complete characterization of the detailed detonation wave structure and physical processes. The detailed multi-step multi-component H_2_-O_2_ combustion mechanism used herein accounts for the key physical processes of OH* formation, emission, and collisional quenching, with the emission rate assumed to be equivalent to the OH* chemiluminescence intensity. The above-mentioned methods have been widely applied in existing research [39].

**Figure 8:**
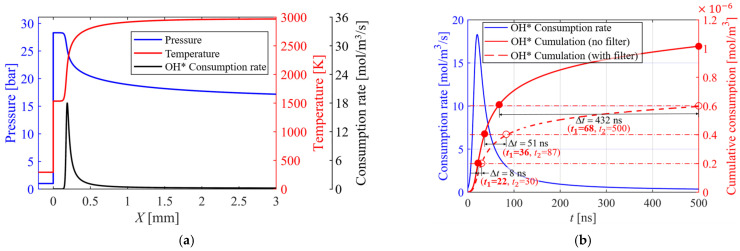
Spatial characteristics of one-dimensional ZND detonation structure in RDC and time evolution characteristics of OH* chemiluminescence: (**a**) profiles of pressure, temperature and OH* consumption rate in H2-air ZND detonation; (**b**) time evolution of OH* consumption rate and cumulative consumption with and without bandpass filter.

Figure 8a shows the calculated one-dimensional ZND detonation structure, including the pressure, temperature, and OH* emission rate profiles. In the ZND framework, the leading shock of the detonation wave compresses the upstream reactant mixture to a high pressure and temperature, as reflected in the plateau region of the pressure and temperature profiles. Following sufficient radical accumulation, violent chain reactions occur, marked by a sharp temperature rise after the plateau and a gradual pressure drop driven by expansion. The OH* emission rate rises markedly in this reaction stage, peaks synchronously with the heat release, and then drops rapidly to a negligible level as the reaction subsides. These results confirm that OH* is confined almost entirely to the high-temperature exothermic main reaction zone, with a spatial distribution strictly aligned with the combustion reaction front. The strong spatial inhomogeneity of OH* in the detonation flow field allows its chemiluminescence intensity to be used for the accurate localization of the reaction zone and the quantitative characterization of the combustion intensity.

Based on the calculation results in Figure 8a, the spatial profiles of the relevant physical parameters are converted to time-domain results via the detonation wave speed, to characterize the time evolution of the parameters at a fixed spatial location. The calculated cumulative OH* emission consumption is shown in Figure 8b, where the blue profile denotes the OH* emission rate, and the red solid and dashed lines represent the cumulative consumption without and with consideration of the remaining ratio of the total OH* chemiluminescence intensity through the filter, respectively. The OH* emission rate rises rapidly to its peak within 20–30 ns before decaying sharply, leading to a two-stage evolution of the cumulative consumption curve: an initial rapid rise followed by a slow increase. Most of the OH* chemiluminescence emission is contributed in the short time window immediately after the shock wave, with the subsequent slow growth dominated by the weak luminescence tail in the late reaction stage (i.e., the low-intensity emission from the near-completion reaction or near-equilibrium region). Further comparison shows that accounting for the filter characteristics leads to a marked reduction in the cumulative OH* emission consumption, with the value at 500 ns reaching only ~60% of that without the filter. For the filter-free case, *t*_1_ = 22 ns, 36 ns, and 68 ns are required to reach 20%, 40%, and 60% of the 500 ns cumulative consumption, respectively; with the filter included, *t*_2_ = 30 ns, 87 ns, and 500 ns are needed for the same cumulative consumption, giving corresponding time differences Δ*t* of 8 ns, 51 ns, and 432 ns. This nonlinearly increasing Δ*t* directly reflects the required GW extension and aggravated time-integration effect caused by the filter, which stems from the coupling between the strong temporal localization of OH* and the filter-induced signal attenuation. While improving the quantum efficiency of the image intensifier or the filter transmission characteristics can theoretically mitigate the adverse impact of signal attenuation on the sensing results, it requires major breakthroughs in the material system and structural process optimization of photocathodes and optical coatings, posing substantial technical challenges.

The above analysis has a direct guiding value for the chemiluminescence sensing of rotating detonation flow fields. In real experiments, it is difficult to simultaneously achieve high-SNR signal acquisition and mitigate the adverse effects of temporal integration. When focusing on the wavefront structure, the relevant analysis can be performed based on the full-spectrum intensity distribution without using a bandpass filter. Although this approach is subject to interferences from metallic flame coloration reactions, near-infrared broadband radiation and other sources, the low temporal-integration effect enabled by a short GW delivers significant advantages in resolving the fine spatial structure of the flow field. On the other hand, when focusing on the heat release distribution characteristics of the flow field, an optical filter is required to eliminate spectral contamination from other wavebands. Although extending the GW to ensure a sufficient SNR will introduce more pronounced temporal-integration effects, the strong correlation between OH* chemiluminescence and chemical reaction heat release allows for the accurate sensing of the flow field heat release characteristics within an acceptable temporal integration range [40,41,42,43,44]. This provides a solid basis for subsequent qualitative or quantitative analysis, and forms complementary advantages with the short-GW scheme.

## 4. Experimental Application in Rotating Detonation Combustion

We tested the HUOSS on a rotating detonation combustor (RDC) experimentally. The system exhibited an excellent performance in high-resolution, high-frame-rate, and flexible multi-band sensing, which will be analyzed in detail in the following sections.

### 4.1. Rotating Detonation Experiment System

**Figure 9:**
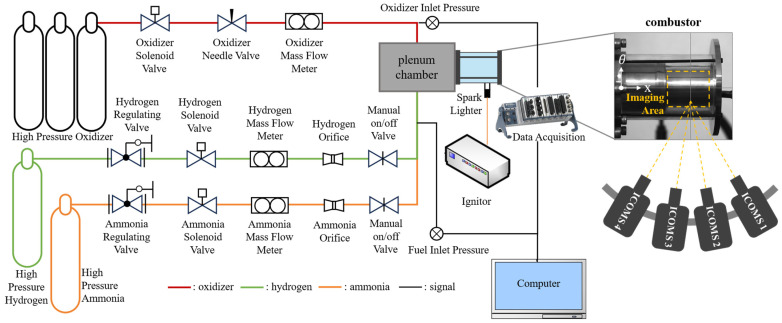
Schematic diagram of the experiment system.

The experimental system of RDC used in this study comprises a gas supply system, a data acquisition system and ignition device, as shown in Figure 9. The oxidizer used in the experiments is the oxygen-enriched air with an oxygen volume fraction of 30%, and the fuels used in the experiments are hydrogen (H_2_) or a hydrogen/ammonia mixture (H_2_/NH_3_). The mass flow rates (MFR) of the oxidizer, hydrogen and ammonia are measured by three mass flow meters (K200S, F505S and F025S, respectively, Emerson Electric Co., St. Louis, Missouri, USA). A high-speed data acquisition instrument (NI cDAQ) is used to collect sensor signals and control the experiment time sequence. The arrangement of the cameras and the shooting area are shown on the left side of Figure 9.

**Figure 10:**
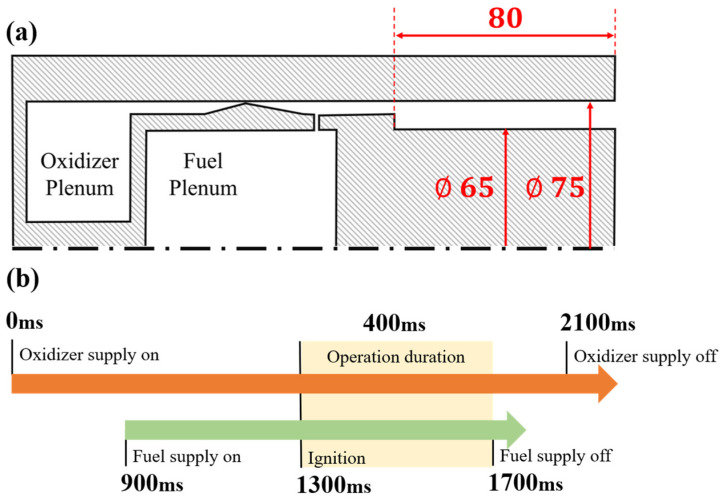
(**a**) Combustor structure; (**b**) Experimental operation timing sequence.

The combustor used in this study has an annular cross-section with the outer diameter *D*_1_ = 75 mm, the inner diameter *D*_0_ = 60 mm, and a length of combustor is 80 mm. The outer wall of the combustor is made of optical quartz glass (ZS-2 conforming to Chinese industry standard JC/T 185-2013 [45], which has an effective transmission wavelength range of 220–2500 nm). The oxidizer enters the annular combustor along the axial direction via a converging-diverging channel, and the fuel enters the combustor radially through injection holes that are arranged along the circumferential direction of the combustor, as shown in Figure 10a. And Figure 10b illustrates the time sequence of the experiment. The operation duration of the combustion device was set at 400 ms, to acquire sufficient valid data and protect the outer wall of the glass combustor from damage. Although the system cannot reach thermal equilibrium within 400 ms, this duration is sufficient to form a stable wave evolution, which is adequate for investigating the flow field structure inside the combustor.

### 4.2. Detonation Wave Structure Resolution—High Resolution of HUOSS

**Figure 11:**
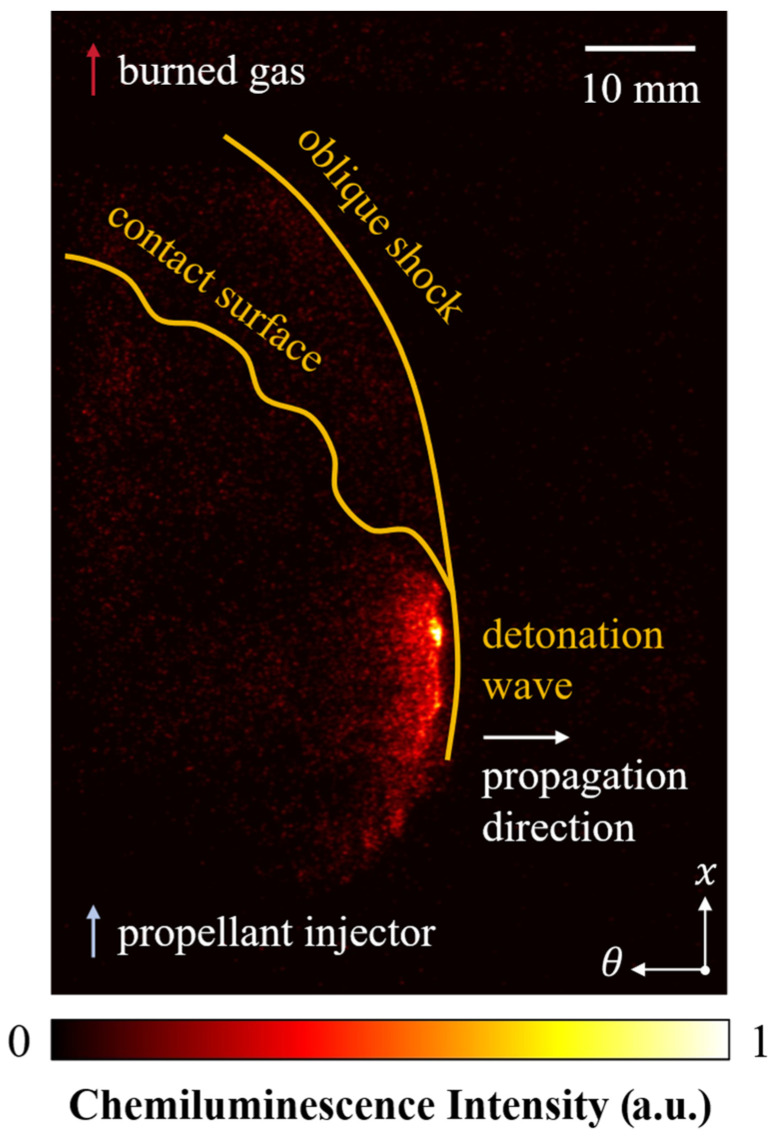
Typical structure of H_2_-air rotating detonation wave (air MFR = 145.42 g/s, equivalence ration (ER) = 0.70).

In a typical RDC, the fuel and oxidizer are separately injected into the combustor through different annular orifices. After the mixture is ignited, the detonation wave, accompanied by its oblique shock wave and contact surface, propagates continuously in the circumferential direction of the combustor, thereby generating a high thrust output. Using the single ICMOS unit mentioned above with an intensifier GW of 200 ns, we obtained the typical flow field of a rotating detonation wave in hydrogen–oxygen mixtures as shown in Figure 11. It should be noted that the experimental images presented in this work underwent the necessary preprocessing and geometric corrections before analysis and visualization. Perspective correction based on manually selected calibration points and homography transformation was applied to compensate for the geometric distortion caused by the different imaging perspectives and optical paths among the ICMOS units. In addition, image undistortion based on camera intrinsic calibration parameters and spatial alignment among different imaging channels were performed to improve the spatial consistency in multi-ICMOS collaborative imaging. Pseudo-color rendering was used for visualization enhancement and did not alter the relative grayscale distribution of the original chemiluminescence images.

In Figure 11, the reactant gases are injected into the combustor along the x-direction from the bottom, and the detonation wave formed after ignition, coupled with oblique shock waves and contact surfaces, propagates along the *θ*-direction, leaving a high-pressure region behind. The figure clearly shows the typical multi-wave coupling structure of the rotating detonation wave. The detonation wave corresponds to the region with the highest chemiluminescence intensity in the flow field, representing the core zone of intense chemical reactions. The red luminous region downstream of the detonation wave and oblique shock waves is the high-temperature and high-pressure product zone after the completion of the chemical reactions, with a slightly lower chemiluminescence intensity than the reaction zone. From the images, the thickness of the detonation reaction zone is estimated to be approximately 1–2 mm, which is consistent with the “thin-layer” characteristic of the detonation reaction zone.

### 4.3. Detonation Wave Evolution—High Frame Rate of HUOSS

Although high-resolution static structures of detonation waves have been obtained, the self-sustaining propagation of rotating detonation waves is essentially an unsteady dynamic equilibrium coupled with multiple processes. Static imaging cannot capture the generation, evolution, abrupt change, and annihilation of the wave system. Therefore, it is necessary to carry out a dynamic process analysis of the detonation waves to further reveal the spatiotemporal evolution laws of the detonation waves.

#### 4.3.1. Experimental Imaging Performance at Different Gate Width

**Figure 12:**
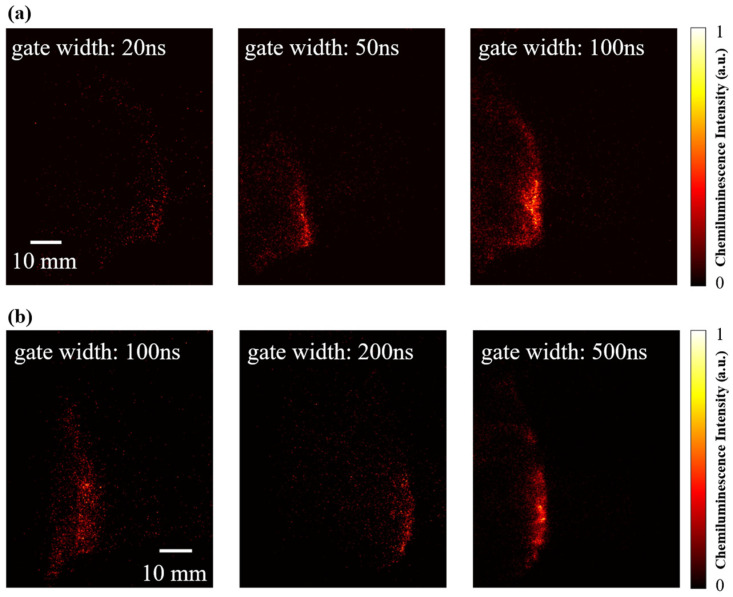
Flow field images of H_2_-air detonation waves under different gate width: (**a**) without the filter (air MFR = 110 ± 5 g/s, ER = 1.00 ± 0.05); (**b**) with the filter (air MFR = 115 ± 5 g/s, ER = 0.88 ± 0.05).

Based on the analysis results in Section 3.2, the flow field images of the RDC have been acquired with different GWs in experiments, and the image quality is evaluated from two perspectives: the image SNR and the temporal integration effect. The results are shown in Figure 12, where (a) and (b) represent the images acquired without and with the filter, respectively. Firstly, when the GW is too short, the structural outline of the detonation wave is indistinct, with the most discrete luminous spots. The noise is relatively pronounced due to the weak signal intensity. As the gate width increases, the detonation wave structure grows progressively clearer, the image noise is reduced and the SNR is markedly improved, while inevitably exacerbating the temporal-integration effect. In addition, a combined analysis of Figure 8 and Figure 12 reveals that for the images acquired without the filter, a high SNR can be achieved with a GW ≥ 50 ns. Taking the pixel cumulative OH* emission consumption at 500 ns in Figure 8 as the reference, the corresponding level at 50 ns can reach approximately 50% of the reference value. In contrast, based on the theoretical calculation results in Figure 8, a GW ≥ 200 ns is required to achieve the same pixel cumulative OH* emission consumption level when filter transmission loss is introduced. As evidenced by the images acquired with the filter shown in Figure 12b, a GW ≥ 200 ns is needed in actual experiments to obtain a favorable SNR. These experimental results verify the validity of the GW analysis presented in Section 3.2, and demonstrate that the introduction of the filter transmission loss exerts a significant impact on the gate width setting. When a filter is employed to investigate the heat release distribution characteristics of the detonation flow field, it is necessary to sacrifice a certain degree of flow field structure resolution in exchange for sufficient OH* chemiluminescence signal intensity, thereby ensuring a favorable SNR.

These OH* chemiluminescence images at different GWs intuitively reveal the decisive influence of the GW on the imaging quality of detonation waves: ultra-short GWs preserve instantaneous fine structures but yields weak signals; a moderate GW balances the resolution and signal-to-noise ratio; and long GWs provide the strongest signals but introduces significant temporal-integration effects. The results indicate that, for the microsecond-scale transient characteristics of H_2_-air detonation, when investigating the macroscopic propagation direction, and overall morphology, the GW should be controlled within 50–100 ns. And when investigating the heat release distribution of the detonation waves, the GW should be controlled within 200–500 ns.

#### 4.3.2. Dynamic Process Analysis of Detonation Waves

**Figure 13:**
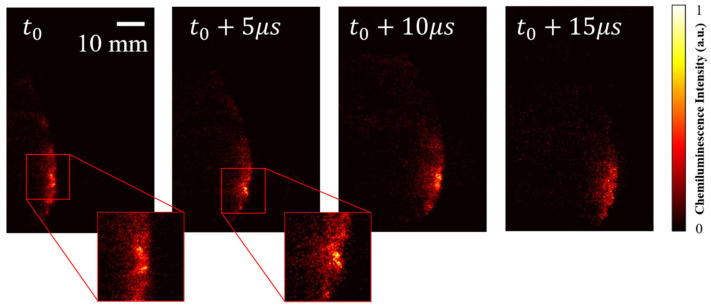
OH* chemiluminescence of H_2_-air detonation waves captured at 5 μs intervals (air MFR = 131.99 g/s, ER = 0.76).

Figure 13 shows a sequence of consecutive OH* chemiluminescence images with a GW of 500 ns and an interval time of 5 μs (equivalent to a frame rate of 200 kHz). The detonation wave propagates from left to right, and the OH* luminous region gradually moves to the right over time. From the time interval, the propagation velocity can be estimated to be approximately 2000 m/s, which is slightly lower than the theoretical CJ velocity (*V*_CJ_ = 2357.9 m/s), reflecting the velocity deficit effect caused by engineering factors such as boundary layers and mixing delays.

Discrete high-intensity hot spots can be observed in the magnified region, which provide visual evidence of detonation cells, reflecting the interaction between the incident shock and transverse waves. Meanwhile, from the overall view of the four images, the positions of the hot spots dynamically evolve over time, demonstrating the unsteady three-dimensional feature of the detonation wave, rather than an ideal planar wave structure. The non-uniform distribution of luminous intensity indicates spatial fluctuations in the heat release rate, which is an intuitive manifestation of detonation instability.

**Figure 14:**
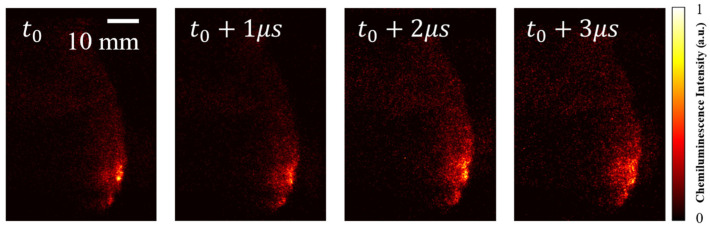
Chemiluminescence of H_2_-air detonation waves captured at 1 μs intervals (air MFR = 145.42 g/s, ER = 0.70).

By narrowing the time interval, the theoretical frame rate of this camera system is greater than 1 MHz. As shown in Figure 14, for example, the evolution of the detonation wave with an interval time of 1 μs (GW 200 ns, without a filter) is presented. At this time scale, the detonation wave under this operating condition exhibits a stable single-wave mode.

### 4.4. Chemiluminescence of Different Radicals—Multi-Band Imaging Capability of HUOSS

**Figure 15:**
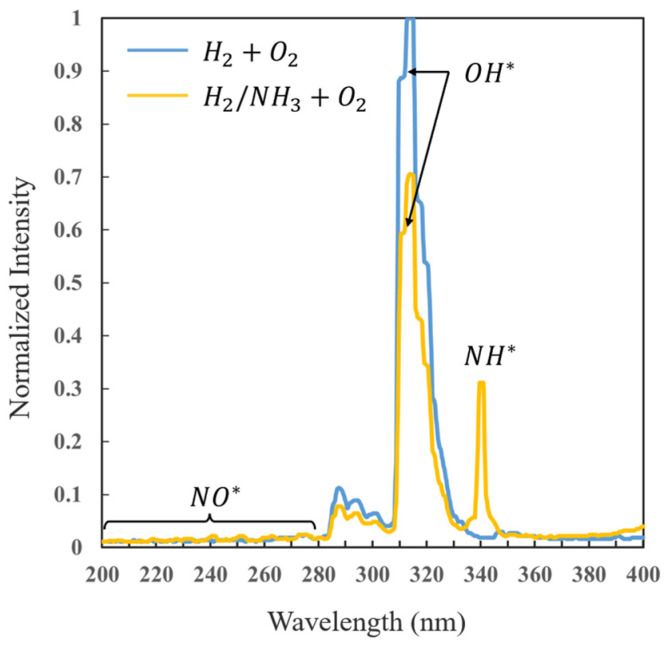
Spectra of rotating detonation combustion [30].

In addition to OH* chemiluminescence, thanks to the flexibility and adaptability of the HUOSS, we can also capture the chemiluminescence of different radicals, enabling multi-component, multi-stage, and multi-scale simultaneous visualization of the detonation flow field. This breaks through the limitation of single-band imaging, which can only observe a single reaction zone. Figure 15 shows the normalized ultraviolet spectra [30], which present the chemiluminescence characteristics of two fuel systems (H_2_-air versus H_2_/NH_3_-air) in the rotating detonation flow field. Both systems exhibit a strong characteristic peak of OH*, which serves as the core tracer signal for the heat release zone in rotating detonation. The H_2_-air system shows a higher peak intensity and a sharper peak shape, while in H_2_/NH_3_-air system, the OH* peak intensity is significantly reduced (to about 0.7), indicating that the addition of NH_3_ dilutes the reactivity of H_2_ and introduces competing reaction pathways, thereby suppressing OH* formation. A distinct characteristic peak of NH* appears only in the H_2_/NH_3_-air system, with an intensity of about 0.3, corresponding to the NH* transition. This provides direct spectral evidence that ammonia participates in combustion and initiates nitrogen chemistry. Both systems show a weak background signal of NO*, implying that NO production is low and occurs mainly in the afterburning or side reaction stage.

**Figure 16:**
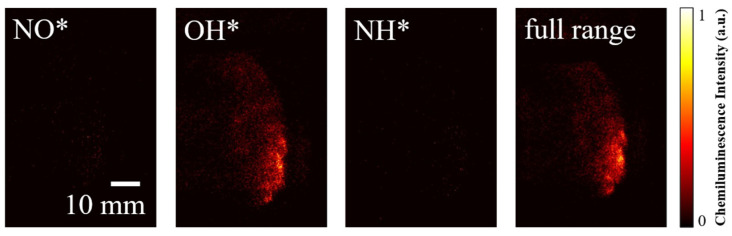
Chemiluminescence of H_2_-air detonation waves at different wavebands (air MFR = 126.12 g/s, ER = 0.87).

**Figure 17:**
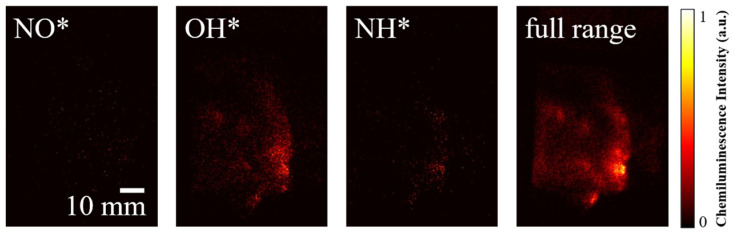
Chemiluminescence of H_2_/NH_3_-air detonation waves at different wavebands (air MFR = 132.66 g/s, ER = 0.81, volume fraction of NH_3_ in the H_2_/NH_3_ mixture = 10.41%).

Figure 16 and Figure 17 show the chemiluminescence images of the H_2_-air rotating detonation flow field and the H_2_/NH_3_-air rotating detonation flow field using different filters: Edmund #67742 (CWL: 254 +10.0/−0.0 nm, FWHM: 40.00 ± 8 nm, Edmund Optics Inc., Barrington, New Jersey, USA) for isolating NO* chemiluminescence, ASAHI ZBPA310 for isolating OH* chemiluminescence, Edmund #65189 (CWL: 337 ± 2.0 nm, FWHM: 10.00 ± 2.0 nm) for isolating NH* chemiluminescence, and no filter for full-spectrum band images, respectively. The GW is set to 500 ns for all the ICOMSs. In Figure 16, the NO* signal is extremely weak, appearing only as discrete noise points, indicating that there is almost no characteristic emission in this band during H_2_-air detonation, which is mainly background interference. This is consistent with the results shown in Figure 15. The OH* signal is the strongest, exhibiting a clear detonation front structure. Local high-intensity hot spots are concentrated at the wavefront, which perfectly marks the reaction zone, consistent with the typical characteristics of H_2_-air detonation: strong shock-reaction coupling and thin-layer heat release. The NH* signal is weak and scattered without an obvious wave front structure, indicating that there is almost no nitrogen-containing radical emission in the pure H_2_-air system, with only a small amount of background noise. The contour of the full-band image is highly consistent with that at 310 nm, and the emission is concentrated at the detonation front, demonstrating that the chemiluminescence of H_2_-air detonation is mainly contributed by OH*, and the total heat release region highly coincides with the reaction zone.

In Figure 17, when ammonia is added, the NO* remains dominated by background noise, but its signal density is slightly higher than that in the pure H_2_-air system, which may originate from the weak emission of trace nitrogen-containing radicals produced by ammonia decomposition. OH* still exhibits a clear detonation front structure, but the emission intensity is weakened, and the distribution becomes more diffuse, indicating that the addition of ammonia dilutes the reactivity of H_2_ and introduces additional reaction pathways, resulting in a reduced OH* generation efficiency and a broadened reaction zone. The NH* signal is significantly enhanced with the addition of ammonia, showing local high-brightness regions corresponding to the detonation wave. This is direct evidence that nitrogen-containing components participate in combustion, marking the region of ammonia decomposition → NH generation → nitrogen-chemistry afterburning, which is spatially complementary to the OH* reaction zone. The full-band emission region is wider and more complex in morphology, including both the leading reaction zone of OH* and the afterburning zone at 337 nm. This demonstrates that the chemiluminescence of H_2_/NH_3_-air detonation is a superposition of emissions from OH* and nitrogen-containing radicals, and the flow field structure exhibits multi-region characteristics. From the above comparison, it can be seen that multi-band imaging enables a quantitative distinction between the heat release zone and the afterburning zone, providing direct experimental evidence for verifying the chemical reaction mechanism of nitrogen-containing fuel detonation, identifying the combustion pathways of nitrogen-containing fuels, and evaluating combustion completeness.

## 5. Conclusions

Extreme combustion is characterized by strong unsteadiness, significant spatial non-uniformity, and intense multi-physics coupling. Existing sensing techniques for such flows face three core challenges: the difficulty in achieving simultaneous multi-spectral observation, high spatiotemporal resolution imaging of ultra-transient processes, and SNR detection of weak signals under short-GW and high frame-rate conditions. To address these limitations, this study developed the HUOSS, which constructs a sensing cluster with a self-developed cost-effective ultraviolet high-gain ICMOS unit as the core component, for the sensing and investigation of extreme combustion processes. The system was successfully applied to the sensing of hydrogen/ammonia-air rotating detonation combustion flow fields, and its multi-spectral, ultra-transient, high-SNR sensing capabilities have been systematically verified. This provides an effective non-intrusive sensing method for elucidating the mechanisms of extreme transient combustion. The specific conclusions are as follows:

- The self-developed ICMOS unit delivers an electron gain of 10^7^ magnitude in the UV band through a double-stage V-stack MCP structure. Based on the advantages of the compact size and cost-effectiveness of the ICMOS unit, this study proposes a design concept for sensing clusters, overcoming the performance limitations of a single ICMOS unit in imaging frame rates and simultaneous multi-spectral detection. In this work, four ICMOS units were integrated specifically, matched with a high-precision timing synchronization control and a PMT trigger module, ultimately realizing megahertz-level imaging at 1608 × 1104 full-frame resolution, which establishes the hardware basis for high-spatiotemporal-resolution diagnosis of extreme combustion flow fields.
- For the chemiluminescence sensing of hydrogen-fueled detonation flow fields, a detailed analysis was conducted on the mechanism by which the two strongly coupled core parameters of the HUOSS, the bandpass filter and the intensifier gate width, affect the performance of the sensing images. While the selected filter preserves ~60% of the total OH* radiation intensity under typical working conditions and ensures the spectral specificity of the target signal, it introduces a transmission loss that significantly affects the gate width setting. Furthermore, we elucidate how the interplay between spatially localized radical emission and signal’s time-integrated results ensures imaging quality in ultra-transient combustion processes. Two complementary imaging schemes are thus established: for investigations focusing on the fine structure of the detonation wavefront, a filter-free scheme with a short GW should be used to achieve high-resolution imaging with low temporal-integration effects; for studies focusing on the heat release characteristics of the flow field, a filter-equipped scheme with a long GW should be used to enable accurate sensing through the strong correlation between OH* chemiluminescence and heat release. These conclusions provide a quantitative foundation for high-speed combustion optical diagnosis
- The HUOSS was applied to the sensing of hydrogen/ammonia-oxygen rotating detonation combustion flow fields, fully validating the comprehensive performance of the system has been. The system clearly resolved the typical multi-wave coupling structure of hydrogen-oxygen rotating detonation waves, demonstrating its high-spatial-resolution imaging capability. With the precise timing control of the multiple ICMOS units, the continuous imaging with an equivalent frame rate ranging from 200 kHz to 1 MHz was achieved, and the propagation and evolution dynamics and unsteady three-dimensional features of detonation waves were captured, which verifies the ultra-transient high-frame-rate imaging performance of the system. Furthermore, the simultaneous multi-band chemiluminescence imaging of OH*, NH* and NO* was performed, revealing the differences in spectral features between the hydrogen-oxygen and hydrogen/ammonia-oxygen rotating detonation combustion systems, and proving the capability of the system for simultaneous multi-spectral sensing.

The HUOSS designed and developed in this study breaks through the performance bottlenecks of existing sensing systems for extreme combustion in simultaneous multi-spectral detection, high spatiotemporal resolution imaging, and high-gain weak signal detection. In the present work, the HUOSS mainly operates in the ultraviolet and visible spectral bands. Specifically, OH*, NH* and NO* chemiluminescence bands are selected for different diagnostic objectives. Benefiting from the modular sensing architecture, the system can also be flexibly extended to other wavelength bands or adapted to different types of photoelectric detectors by replacing the corresponding optical and detection components according to specific diagnostic requirements. Meanwhile, the system is not only suitable for rotating detonation combustion flow fields, but it also can be extended to research on other extreme combustion scenarios such as internal combustion engines and scramjet engines, which provides comprehensive experimental data support for the optimization of combustion organization and performance enhancement of advanced power devices.

## Figures and Tables

**Table 1:** Recent applications of UV chemiluminescence under extreme combustion.

Application	Cameras/Intensifier	Parameters Integration Time/Frame Rate/Resolution/Response Band	Research
deflagration and rotating detonation	Shimadzu HPV-X2/Lambert Instruments HiCatt	110 ns (min)/5 MHz (max)/400 × 250/-	Wang R. B., 2024 [17]
linear detonation channel	Phantom v2512/Lambert HiCatt S20	265 ns (min)/100 kHz /20 × 512 /~270–450 nm	Ayers Z. M., 2022 [22]
ramjet mode in RBCC engine	PCO dimax HS1/self-developed models intensifiers	125 μs/7.145 kHz /0.184 mm/pixel /-	Zhang D., 2026 [26]
scramjet model combustor with two-stage fuel injection	PHotron Fastcam NOVA S20/C4412, Hamamatsu Photonics K.K. and C4273, Hamamatsu Photonics K.K.	1.1 μs (min)/7.5 kHz /1024 × 1024 (max) /~1300–7200 nm	Norimatsu K., 2026 [27]
dual fuel Hydrogen/Diesel combustion in a single cylinder research engine	Photron Fastcam SA-X2/LaVision’s High-Speed IRO	45 μs/10 kHz/ 515 × 512/~190–650 nm	Rossetti S., 2025 [28]
high-pressure liquid ammonia spray combustion	Photron Mini AX-200/IIM-C, Zolix	1 μs (min)/10.8 kHz/ 173 μm/pixe/ ~200–850 nm	Wu H., 2025 [29]

## Data Availability

The data presented in this study is included in the article.

## Abbreviations

The following abbreviations are used in this manuscript:

HUOSS	High-performance ultraviolet optical sensing system
PMT	Photomultiplier tube
UV	Ultraviolet
UV-PLIF	Ultraviolet planar laser-induced fluorescence
GW	Gate width
SNR	Signal-noise-ratio
ICOMS	Intensified complementary metal oxide semiconductor
CMOS	Complementary metal oxide semiconductor
MCP	Microchannel plate
CWL	Central wavelength length
FWHM	Full width at half maximum
RDC	Rotating detonation combustor
MFR	Mass flow rates
ER	Equivalence ratio

## References

[1] Tong Z., Wu H., Shi Z., Myagkov L. (2025). A review on the influence of in-cylinder turbulence type on combustion characteristics of high power-density diesel engine. J. Traffic Transp. Eng. (Engl. Ed.).

[2] Geng L., Xiao H., Cui Y., Gao N., Li Q., Chen H., Xie Y. (2023). Numerical study of effect of post injection coupled with EGR on combustion and emission performances of CRDI engine fueled with biodiesel-ethanol blends. J. Traffic Transp. Eng. (Engl. Ed.).

[3] So Khuong L., Hashimoto N., Fujita O. (2024). Spray, droplet evaporation, combustion, and emission characteristics of future transport fuels for compression-ignition engines: A review. J. Traffic Transp. Eng. (Engl. Ed.).

[4] Guan Y., Becker S., Zhao D. (2025). Research and Development on Ramjet Combustion Instabilities. J. Therm. Sci..

[5] Shepherd J.E. (2009). Detonation in gases. Proc. Combust. Inst..

[6] Ivanov V.S., Frolov S.M., Zangiev A.E., Zvegintsev V.I., Shamshin I.O. (2022). Updated conceptual design of hydrogen/ethylene fueled detonation ramjet: Test fires at Mach 1.5, 2.0, and 2.5. Aerosp. Sci. Technol..

[7] Ma J.Z., Luan M.-Y., Xia Z.-J., Wang J.-P., Zhang S.-j., Yao S.-b., Wang B. (2020). Recent Progress, Development Trends, and Consideration of Continuous Detonation Engines. AIAA J..

[8] Raman V., Prakash S., Gamba M. (2023). Nonidealities in Rotating Detonation Engines. Annu. Rev. Fluid Mech..

[9] Wang B., Xie Q.F., Wen H.C., Teng H.H., Zhang Y.N., Zhou L. (2021). Research Progress of Detonation Engines. Tuijin Jishu/J. Propuls. Technol..

[10] Ding C., Wu Y., Huang Y., Zheng Q., Li Q., Xu G., Kang C., Weng C. (2023). Wave mode analysis of a turbine guide vane-integrated rotating detonation combustor based on instantaneous frequency identification. Energy.

[11] Sandri U., Ramanagar Sridhara S., Andreini A., Bohon M., Picchi A., Facchini B. (2023). Evaluation of Cooling Requirements for Rotating Detonation Combustors. J. Turbomach..

[12] Su L., Wen F., Wang S., Wang Z. (2022). Analysis of Energy Saving and Thrust Characteristics of Rotating Detonation Turbine Engine. Aerosp. Sci. Technol..

[13] Anand V., St. George A., Driscoll R., Gutmark E. (2015). Characterization of instabilities in a Rotating Detonation Combustor. Int. J. Hydrogen Energy.

[14] Jiao Z., Wang K., Xiao Q., Zhang Y., Fan W. (2024). Characteristic velocity analysis of the total pressure gain of rotating detonation combustors. Proc. Combust. Inst..

[15] Bu M., Wen H., Dai W., Xie Q., Shi Y., Wang B. (2026). Rotating detonation bladeless turbine prototype: An experimental analysis of combustion characteristics and power output. Aerosp. Sci. Technol..

[16] Lu H., Lin J., Jin Y., Chen X., Ji F., Wu H., Liu C., Wang R., Zhu H., Yang F. (2024). Hydrogen combustion experiments of a fullpath scramjet at a Mach 10 condition of high enthalpy shock tunnel. Acta Aerodyn. Sin..

[17] Wang R.B., Webb A.M., Athmanathan V., Slipchenko M.N., Kearney S.P., Perkins H.D., Roy S., Fugger C.A., Meyer T.R. (2024). 500-kHz OH PLIF and OH* chemiluminescence imaging of deflagration and rotating detonation in CH4-O2 and H2-air mixtures. Proc. Combust. Inst..

[18] Wu G., Yu X., Peng J., Yang C., Cao Z., Qin F., Zhu S., Yuan X., Zhang S., Chen X. (2025). Experimental investigation on the mixing and combustion characteristics with dual combined flame stabilizers in scramjet using simultaneous OH/kerosene-PLIF and Mie scattering measurements. Appl. Therm. Eng..

[19] Somarathne K.D.K.A., Okafor E.C., Sugawara D., Hayakawa A., Kobayashi H. (2021). Effects of OH concentration and temperature on NO emission characteristics of turbulent non-premixed CH4/NH3/air flames in a two-stage gas turbine like combustor at high pressure. Proc. Combust. Inst..

[20] Wang S., Hanson R.K. (2018). High-sensitivity 308.6-nm laser absorption diagnostic optimized for OH measurement in shock tube combustion studies. Appl. Phys. B.

[21] Liang S., Zhang Z., Ding H., Ma X., Li Y., Xu H. (2018). Mixture Distribution Measurement in the Tumble Plane of a GDI Engine via PLIF Method. Neiranji Xuebao/Trans. CSICE (Chin. Soc. Intern. Combust. Engines).

[22] Ayers Z.M., Lemcherfi A., Plaehn E.W., Gejji R.M., Perkins H.D., Roy S., Slabaugh C.D., Meyer T.R., Fugger C.A. (2022). Simultaneous 100-kHz acetone planar laser-induced fluorescence and OH* chemiluminescence in a linear non-premixed detonation channel. Combust. Flame.

[23] Hoeper M.W., Webb A.M., Athmanathan V., Wang R.B., Douglas Perkins H., Roy S., Meyer T.R., Fugger C.A. (2023). Liquid fuel refill dynamics in a rotating detonation combustor using megahertz planar laser-induced fluorescence. Proc. Combust. Inst..

[24] Rankin B.A., Fugger C.A., Richardson D.R., Cho K.Y., Hoke J., Caswell A.W., Gord J.R., Schauer F. (2016). Evaluation of Mixing Processes in a Non-Premixed Rotating Detonation Engine Using Acetone PLIF. Proceedings of the 54th AIAA Aerospace Sciences Meeting, San Diego, CA, USA, 4–8 January 2016.

[25] Zhou Y., Bai Y.H., Song X.D., Yao M., Wang J.F., Su W.G., Yu G.S. (2020). Application of Chemiluminescence in Spectral Diagnosis: A Review. Spectrosc. Spectr. Anal..

[26] Zhang D., Yang K., Li P., An B., Sun M., Wang T., Liang C., Li M., Wang J., Xie Y. (2026). Flame stabilization and enhancement mechanisms assisted by hydrogen micro-jet in ramjet mode of RBCC engine under low flight Mach number. Combust. Flame.

[27] Norimatsu K., Katsumura N., Nishiura S., Kudo T., Hayakawa A. (2026). Pressure profiles and flame behaviors of a scramjet model combustor with two-stage fuel injection for low flight Mach numbers. Acta Astronaut..

[28] Rossetti S., Mancaruso E., Montanaro A., Vaglieco B.M. (2025). Analysis of dual fuel Hydrogen/Diesel combustion in ultra lean conditions via simultaneous UV and IR imaging. Appl. Therm. Eng..

[29] Wu H., Qian Y., Zhang T., Zhu J., Lu X. (2025). Ignition and flame development of high-pressure liquid ammonia spray combustion with simultaneous high-speed OH* and NH2* chemiluminescence imaging. Combust. Flame.

[30] Ma J., Dai W., Chen D., Hu J., Yang D., Wang L., Zheng D., Shi Y., Wen H., Wang B. (2025). A Nanosecond-Scale, High-Spatiotemporal-Resolution, Near-UV–Visible Imaging System for Advanced Optical Diagnostics with Application to Rotating Detonation Engines. Photonics.

[31] Ma J., Fu X., Chen D., Chang L., Wang L., Shi Y., Wen H., Wang B. (2026). UV and Visible Radiation Characteristics of Thermoacoustic Instabilities in an Ammonia–Methane Premixed Swirl-Stabilized Combustor. Energies.

[32] Bass M. (2009). Handbook of Optics, Third Edition Volume II: Design, Fabrication and Testing, Sources and Detectors, Radiometry and Photometry.

[33] Fujiwara T., Takeuchi S., Kalay Z., Nagai Y., Tsunoyama T.-A., Kalkbrenner T., Iwasawa K., Ritchie K., Suzuki K., Kusumi A. (2023). Development of ultrafast camera-based single fluorescent-molecule imaging for cell biology. J. Cell Biol..

[34] Feng X.O., Jin Y., Zhai C. (2023). Summary of research on flame 3D reconstruction based on computed tomography of chemiluminescence technology. J. Exp. Fluid Mech..

[35] Wang H., Guo J., Miao J., Luo W., Gu Y., Xie R., Wang F., Zhang L., Wang P., Hu W. (2022). Emerging Single-Photon Detectors Based on Low-Dimensional Materials. Small.

[36] Feleo A., Chacon F., Gamba M. (2019). Effects of Heat Release Distribution on Detonation Properties in a H2/Air Rotating Detonation Combustor from OH* Chemiluminesence. AIAA Propulsion and Energy 2019 Forum.

[37] Feleo A., Chacon F., White L.W., Gamba M. (2019). Evaluation of OH Emission for Determining Operation of a Rotating Detonation Engine. AIAA Scitech 2019 Forum.

[38] SRI (1999). LIFBASE: Database and Spectral Simulation Program.

[39] Zhao M., Buttsworth D., Choudhury R. (2018). Experimental and numerical study of OH* chemiluminescence in hydrogen diffusion flames. Combust. Flame.

[40] Escudero F., Fuentes A., Demarco R., Consalvi J.L., Liu F., Elicer-Cortés J.C., Fernandez-Pello C. (2016). Effects of oxygen index on soot production and temperature in an ethylene inverse diffusion flame. Exp. Therm. Fluid Sci..

[41] Hardalupas Y., Orain M. (2004). Local measurements of the time-dependent heat release rate and equivalence ratio using chemiluminescent emission from a flame. Combust. Flame.

[42] He L., Guo Q., Gong Y., Wang F., Yu G. (2019). Investigation of OH* chemiluminescence and heat release in laminar methane–oxygen co-flow diffusion flames. Combust. Flame.

[43] Kathrotia T., Riedel U., Seipel A., Moshammer K., Brockhinke A. (2012). Experimental and numerical study of chemiluminescent species in low-pressure flames. Appl. Phys. B.

[44] Kathrotia T., Riedel U., Warnatz J. A Numerical Study on the Relation of OH*, CH*, and C2* Chemiluminescence and Heat Release in Premixed Methane Flames. Proceedings of the 4th European Combustion Meeting.

[45] (2013). Optical Quartz Glass.

